# The Sources of Muscular Power

**Published:** 1868-03

**Authors:** Frank W. Abbott


ART. II—*The Sources of Muscular Power.* BY FRANK W. ABBOTT, M. D.

The study of the vital forces is of so much interest to members of our profession, that I have presumed to make one branch of it the subject of this paper, although it is a little apart from the topics usually discussed by this Society. Of course in such a subject as this there can be no attempt at originality by one who has but just commenced the general practice of medicine; but the field of our studies is so broad, that the corner where I glean may not have been lately visited by some of you, and so my handful of gathered ears may be as new as though it were a bundle of one of the original reapers.

In the study of the "sources of muscular power," as in all the other natural sciences, sure progress could only be made when the ancient methods of speculation were given up for the critical analyses and rigid demonstrations of modern science; and here, too, the achievements of collateral sciences are essential instruments for progress. The theory of muscular power which we shall endeavor to sustain, rests for its proof upon the theory of the correlation and conservation of forces, and as this is of comparatively recent enunciation, it may be well to consider it more closely for a moment. By the correlation of forces then, we understand that the five forces—one mechanical or molar, viz: motion, and four molecular, viz: heat, light, electricity, and chemical force—are severally interchangeable, and can be transformed each into the other, and that there is a definite, fixed ratio between them.—Indeed the experiments of Tyndall, as detailed in his "Lectures," prove that heat is merely a molecular motion, and the theory of caloric having had its day must take its place with that of phlogiston. By the conservation of force we understand that force is indestructible; if it disappears in one form it re-appears in another, and therefore that as the sum of matter in the universe is the same as on the day of creation, so the sum of force in the universe is the same that it ever has been—none has been created, none destroyed—it merely varies its form and manifestations to suit the varying needs of the universe.

Starting from this point we shall not be satisfied with the text books which the physiologists give us, when we ask what is the source of muscular power. They tell us of a "peculiar force which is generated by living animals," which they call "vital force," and observing that muscular tissue contracts under some stimuli, they say that contractility or "irritability" is an inherent property of muscle, but to all questions as to the source of this vital force they are either silent or return very unsatisfactory answers. Chemists have studied the ingesta and excreta, and from these it was learned that much the greater portion of the food taken into the system consists of substances containing but very little oxygen, but the excreta and exhalations are very highly oxydized. Observing that the oxydation or combustion of matter external to the body was always accompanied by the evolution of force, usually in the form of heat, it was very natural to infer that the *heat* of the body was caused by the oxydation of food. It would be interesting to trace the progress of belief on this subject, but my time is too limited. I must be content with a brief statement of the theory supported by the powerful name of Liebig, and which, as is well known to you all is generally received. He divided food broadly into two classes, viz: albumenoids, or nitrogenous, such as lean meat, eggs, etc., and non-nitrogenous substances, such as the starches, sugars, fats, etc. Of these the nitrogenous alone are assimilated, and enter into the composition of the tissues of the body, while the non-nitrogenous are oxydized without being transformed into tissue, and their oxydation produces heat, and heat alone, and the source of muscular power, or mechanical work of the muscles is the oxydation of the muscular tissue itself. The reasoning seems good; force is neither created nor destroyed; in muscular action force is expended; in the oyxdation of matter force is liberated; we recognize in the the excreta the products of oxydation of muscle, therefore the oxydation of muscle is the source of muscular power. But this theory was put to the test of experiment, and like many another ingeniously wrought theory, it failed to pass the ordeal. There are three data which must be obtained to test this question, which are these:— 1. The amount of mechanical work performed by the muscular system in a given time. 2. The amount of muscle consumed in same time. 3. The quantity of mechanical work which the oxydation of a given amount of muscle is capable of producing.

An experiment to ascertain the first two data for our problem was performed by Profs. Fick and Wiscenlenus of Zurich, who for this purpose climbed the mountain of Faulhorn, whose height is accurately known, and calculated carefully the amount of muscular work required for the ascent. They added to this the estimated amount of work done by the circulatory and respiratory systems. The sum gave them our first wished for datum, the amount of muscular work done in a given time. At the same time they measured the amount of nitrogen secreted in the urea during the ascent, and for six hours after, to allow for the elimination of all the urea produced during the ascent, and from this they calculated the amount of muscle consumed during the ascent, which gave our second datum.

Dr. Frankland, from whose lecture before the Royal Institute of Great Britain, delivered in June, 1866, I have taken the following details and calculations, calculated the amount of work which could be done by the oxydation of this amount of muscle, and found that it was by no means equal to the work done during the ascent.

But to bring this subject more distinctly before you, I will give a brief summary of the details of these experiments, reducing the meters, kilogrammes and meter kilogrammes of Dr. Frankland's lecture to feet, lbs., and foot-lbs. In these calculations we shall use to express the relation between heat and mechanical work the 772 foot-pounds of Joule. He found that the force generated by a pound weight falling 772 feet, if converted into heat, would elevate the temperature of a pound of water one degree, or, conversely, that the heat required to elevate the temperature of one pound of water one degree; if transformed into motion would be sufficient to lift it 772 feet, or, which is the same thing, to lift 772 pounds one foot. He took the force required to lift one pound one foot as his unit of force, and called it a foot-pound, and as we have seen the heat required to elevate the temperature of one pound of water one degree, whether produced by the oxydation of charcoal or muscle, is equal to 772 foot-pounds of mechanical work; if a man weighing 140 pounds climbs a mountain 1000 feet high, 140,000 foot-pounds will be the mechanical work done.

In the experiments referred to, Fick with his equipments weighed 146 lbs., Wiscenlinus 168 lbs.; the heighth of the mountain was 6,418 feet, which being multiplied by the weight of the parties, gives for the external work of Fick 937,028 foot-pounds; of W. 1,078,224 foot-pounds. To this we must add the work performed by the heart, which is estimated at 4.63 foot-pounds for each systole. Fick's heart averaged 120 pulsations per minute; the time of the ascent was about 5½ hours, which would make the force expended by the heart about 183,348 foot-pounds. The force exerted by the muscles of respiration is estimated to be 4.56 foot-pounds for each inspiration. Fick breathed 25 times per minute, and this gives us 37,610 foot-pounds as the muscular force expended in respiration, making the total of internal and external work 1,157,986 foot-pounds. The total internal and external work of W. was estimated to be 1,336,008 foot-pounds, and this is evidently less than the actual amount of work performed, since we have left out of account all other muscular action except the circulation, respiration, and the force necessary to raise the body perpendicularly to the height of the mountain.

The calculation as to the amount of muscle consumed in doing this work is as follows:

| | Fick. | Wiscen. |
|---|---|---|
| Amount of nitrogen excreted during the ascent, | 51 grs. | 50.15 grs. |
| " " " " succeeding 6 hours, | 37.4 " | 37.26 " |
| Total, | 88.4 grs. | 87.41 grs. |
| Amount of dry muscle corresponding to 88.4 & 87.41 grs. nit. | 571.4 grs. | 569.8 grs. |

allowing that 1 gr. of nitrogen represents a little less than 6.5 grs. of dry muscle. This will certainly not seem to be an under estimate, when we consider that at this rate the whole muscular tissue would be consumed in about 46 days in work alone.

Dr. Frankland's beautiful and conclusive experiments to ascertain the actual energy evolved in the combustion of muscle cannot be detailed here; suffice it to say, that he calculated that the actual energy evolved in the combustion of one grain of muscle is equal to 868 foot-pounds of mechanical work; if we multiply this by the grains of muscle consumed, 571.4 for Fick, and 569.8 for Wiscenlinus, we get as the actual energy capable of being produced by the muscle consumed during the ascent for Fick 495,975 foot-pounds, W. 494,586 foot-pounds. We saw that the mechanical work performed by Fick was 1,157,986 foot-pounds, and by W. 1,336,008 foot-pounds; but at the most liberal calculation no more than one-half the actual energy developed in the consumption of muscle appears as mechanical work, (in the best steam engines less than one-tenth of the actual energy developed in the consumption of the fuel appears as work,) the rest taking the form of heat, etc., so it is evident that for these 1,157,986 and 1,336,008 foot-pounds of work, at least 2,315,972 and 2,672,016 foot-pounds of actual energy must have been produced in the body; if we compare this with the amount of actual energy capable of being produced by the amount of muscle consumed, 495,975 foot-pounds for Fick and 494,586 foot-pounds for W., we see that "scarcely one-fifth of the actual energy required for the work could be obtained from the amount of muscle consumed."

The following table is a concise statement of this calculation:

| | Fick. | Wiscenlinus |
|---|---|---|
| Amount of dry muscle consumed, - - - - - - - | 571.4 grs. | 569.8 grs. |
| Actual energy capable of being produced by the consumption in the body of 571.4 and 569.8 grs. of dry muscle, - - - | Foot-pounds 495,975 | Foot-pounds 494,786 |
| Measured work performed in the ascent, (External Work,) - - | * 937,028 | 1,078,224 |
| Estimated work, Circulation, - - - - - - - - | 183,348 | 214,906 |
| " " Respiration, - - - - - - - - | 37,610 | 43,878 |
| Total work performed, - - - - - - - - - | 1,157,986 | 1,336,008 |
| Multiplying by 2, - - - - - - - - - | | X.2 |
| Amount of actual energy required to do this work, - - - | 2,315,972 | 2,672,016 |

The experiments of Smith upon prisoners in a tread-mill, of Houghton upon military prisoners engaged in a shot-drill, and Playfair upon common laborers, although not so thorough as the one detailed, all tend to substantiate the view that the source of muscular power is not to be found solely or even chiefly in the oxydation of the muscular tissue itself. Indeed it seems as though the waste of muscle is not much affected by the amount of work done, not much more nitrogen being secreted during severe labor than during rest.

Our time so far has been taken up in pulling down old theories of the source of muscular power; let us see if we can find a new one which is more satisfactory. Careful observations have shown that the amount of carbonic acid gas exhaled in respiration varies directly with the amount of muscular exertion. In one experiment the following results were obtained:

* Since making these reductions from the Metrical system, I have seen some of the same in print, differing somewhat from mine, but as the most of my reductions were carried out from three to six places of decimals, and a careful repetition has only confirmed their correctness, I have not seen fit to change them.

| | | | | | |
|---|---|---|---|---|---|
| During sleep the amount of carbonic acid gas excreted, per hour, was, grains | | | | | 292.6 |
| Lying down and sleep approaching, | " | " | " | " | 354.2 |
| Sitting down, | " | " | " | " | 446.6 |
| Walking at the rate of 2 miles per hour, | | " | " | " | 1087.8 |
| Walking at the rate of 3 miles per hour, | | " | " | " | 1552.3 |
| Upon a tread-mill ascending 28.65 feet per minute, | | | " | " | 2290.8 |

Hence we see that increased muscular exertion is accompanied by an increased excretion of carbonic acid gas. When we look for the source of this we find that the principal supply of carbon comes from the starches, sugars, and fats taken as food, and as force of some kind must be produced by its oxydation, and as the increased heat of the body during exercise is not sufficient to absorb this surplus force, the most natural supposition is, that the muscular system is in the main a machine in which the force, or actual energy, generated by the oxydation of the non-nitrogenous elements of the blood, which circulates through it, is changed into mechanical work. But accurate observations as to the relation between the amount of work done by the muscles and the amount of carbonic acid gas exhaled from the lungs are needed to establish, or perhaps to overthrow this theory.

In this connection a sentence which occurs in an essay written by Dr. Carpenter, the distinguished physiologist, before these experiments were made, is interesting as showing the drift of thought in this direction. He says "motor force may be developed like heat by the metamorphosis of constituents of food, which are not converted into living tissue; an inference which so fully harmonizes with the doctrine of the direct convertibility of these two forces, now established as one of the surest results of physical investigation, as to have in itself no inherent improbability." And again, "it can scarcely be regarded as improbable that the constant activity of the heart and respiratory muscles, which gives them no opportunity of renovation is sustained not so much by the continual renewing of their substance, (of which renewal there is no histological evidence whatever) as by the metamorphosis of matters external to themselves, supplying a force which is manifested through their instrumentality."

From the table given by Dr. Frankland of the actual energy produced by the oxydation in the body of one gramme of various articles of food, I have calculated the amount of work capable of being produced by the old army ration, with this result:

| | | |
|---|---|---|
| 20 oz. of beef, - | 2,477,415 | foot-pounds. |
| 18 oz. of bread, - - | 3,357,299 | " " |
| 0.15 lbs. of sugar, - | 683,313 | " " |
| 0.16 lbs. of peas, - - | 832,315 | " " |
| Total, - - - - | 7,350,342 | |

The average external work of a soldier is estimated to be about 347,232 foot-pounds per diem; the internal work, i. e. in the circulation and respiration 578,763; total, 925,995 foot-pounds; multiplying this by two, as in the preceding experiments, to get the estimated amount of actual energy required for this amount of mechanical work, we get 1,852,010 foot-pounds of actual energy required, and comparing this with the 7,350,342 foot-pounds of actual energy capable of being produced by his food, it certainly appears to leave sufficient force unemployed for the extra muscular work, such as the peristaltic movements of the intestines, the continual tonic contraction of the muscles, etc., for the heat of the body, the force probably consumed in digestion, assimilation, and (we are conscious that here we tread on very uncertain and perhaps dangerous ground,) the actual energy which may be required to produce nerve force, and even what we are accustomed to call purely intellectual forces, such as volition, perception, reflection, etc.

There are many interesting and practical questions connected with this subject, but fearing lest I may already have taxed your patience with this array of theories, facts and figures, I hasten to a conclusion, thanking you for the honor conferred in choosing me to read this paper, and for the kind attention it has received.

